# Screening of high-efficiency nitrogen-fixing bacteria from the traditional Chinese medicine plant Astragalus mongolicus and its effect on plant growth promotion and bacterial communities in the rhizosphere

**DOI:** 10.1186/s12866-023-03026-1

**Published:** 2023-10-16

**Authors:** Zhiyong Shi, Xu Guo, Zhenhong Lei, Yuanyuan Wang, Zhenyu Yang, Jingping Niu, Jianping Liang

**Affiliations:** 1 College Of Life Sciences, Shanxi Agricultural University, Jinzhong, 030801 China; 2 Shanxi Zhendong Pharmaceutical (China), Changzhi, 047000 China; 3 Shanxi Key Laboratory of Chinese Veterinary Medicine Modernization, Shanxi Agricultural University, Jinzhong, 030801 China

**Keywords:** *Astragalus mongolicus*, PGPR, Biological nitrogen fixation, Plant growth promotion, Targeted amplicon sequencing, Bacterial community

## Abstract

**Background:**

*Astragalus mongolicus* Bunge is used in traditional Chinese medicine and is thus cultivated in bulk. The cultivation of *A. mongolicus* requires a large amount of nitrogen fertilizer, increasing the planting cost of medicinal materials and polluting the environment. Isolation and screening of plant growth-promoting rhizobacteria (PGPR) and exploring the nitrogen fixation potential of *A. mongolicus* rhizosphere microorganisms would effectively reduce the production cost of *A. mongolicus*.

**Results:**

This study used *A. mongolicus* roots and rhizosphere soil samples from Longxi County of Gansu Province, Jingle County, and Hunyuan County of Shanxi Province, China, to isolate and identify nitrogen-fixing bacteria. Through nitrogen fixation efficiency test, single strain inoculation test, and plant growth-promoting characteristics, three strains, *Bacillus sp.* J1, *Arthrobacter sp.* J2, and *Bacillus sp.* G4 were selected from 86 strains of potential nitrogen-fixing bacteria, which were the most effective in promoting the *A. mongolicus* growth and increasing the nitrogen, phosphorus, and potassium content in plants. The antagonistic test showed that these bacteria could grow smoothly under the co-culture conditions. The J1, J2, and G4 strains were used in a mixed inoculum and found to enhance the biomass of *A. mongolicus* plants and the accumulation of the main medicinal components in the field experiment. Mixed bacterial agent inoculation also increased bacterial diversity and changed the structure of the bacterial community in rhizosphere soil. Meanwhile, the relative abundance of Proteobacteria increased significantly after inoculation, suggesting that Proteobacteria play an important role in plant growth promotion.

**Conclusions:**

These findings indicate that specific and efficient PGPRs have a significant promoting effect on the growth of *A. mongolicus*, while also having a positive impact on the structure of the host rhizosphere bacteria community. This study provides a basis for developing a nitrogen-fixing bacterial fertilizer and improving the ecological planting efficiency of *A. mongolicus.*

## Introduction

Traditional agriculture relies heavily on chemical fertilizers and pesticides to improve agricultural productivity to feed the growing world population [1]. These practices are costly and lead to the degradation of arable land, which harms the environment. This is especially true for the overuse of chemical fertilizer [2] as, disregarding the balance between land use and nutrition, plants will only absorb 30-40% of the nitrogen fertilizer [3]. This will result not only in reduced fertility but also in soil acidification, decreased organic matter content [4], water eutrophication [5], and nitrate pollution in groundwater and drinking water [3], causing severe diseases and the proliferation of pests. Therefore, using plant growth-promoting rhizobacteria (PGPR) in agriculture may be a sustainable and environmentally friendly solution to reduce the problems associated with the overuse of chemical fertilizer [6]. PGPR are microorganisms that live in the plant rhizosphere and have been shown to promote plant growth, control diseases, and increase crop yields [7]. PGPR can promote plant growth through direct and indirect mechanisms [8]. Direct mechanisms refer to specific bacterial traits that directly promote plant growth, including the production of auxin, 1-aminocyclopropane-1-carboxylate (ACC), deaminase, cytokinin, gibberellin, nitrogen fixation, phosphorus dissolution, and iron chelation by bacterial iron carriers. Indirect mechanisms are related to bacterial characteristics that inhibit the function of one or more plant pathogenic organisms (fungi and bacteria), including ACC deaminase, antibiotics, cell wall-degrading enzymes, hydrogen cyanide, and induced systemic resistance [9, 10]. In addition to these methods for controlling plant pathogens, PGPR can selectively use phages to biocontrol certain bacterial pathogens [11]. Although PGPR is a common resident in the soil, their numbers are not sufficient to compete with other bacteria established in the rhizosphere. Therefore, it is necessary to inoculate PGPR to increase the number of target microorganisms in the soil and to maximize their beneficial effects on the plant yield.

Bacterial fertilizer using PGPR as the raw material can add a large number of microorganisms to the soil to improve the nutritional environment of crops [12]. Bacterial fertilizer plays an important role in improving soil fertility and the fertilizer utilization rate as it can rapidly proliferate, resulting in increased numbers of beneficial bacteria that provide nutrition for plants, assist in nutrient absorption, promote growth, and enhance plant resistance to pathogens thus reducing both disease and pests [13]. However, the application and development of traditional bacterial agents are restricted by poor environmental adaptability, low inoculation efficiency, and unstable inoculation. In recent years, bacterial fertilizers composed of single-strain and single-function microorganisms have been replaced by fertilizers containing a variety of strains and functions [14].

The efficacy of microbial fertilizers with nitrogen-fixing bacteria as the main constituent is affected by many factors, including crop type, amount of bacterial fertilizer, and soil moisture and organic matter content [15]. Although nitrogen-fixing bacteria are usually present in traditional biological fertilizers and are effective with certain crop varieties, they have limited efficacy due to low matching with the host plant, weak competition with indigenous customized rhizosphere bacteria, and poor adaptability to the soil environment [16]. Therefore, it is necessary to isolate specific nitrogen-fixing bacteria from the host as the active ingredient of the inoculant. Rhizosphere colonization is a prerequisite for PGPR to affect plants [17]. The isolation and study of nitrogen-fixing bacteria and their nitrogen-fixing effect from the host rhizosphere environment will help to improve bacterial fertility and develop specific nitrogen-fixing bacteria fertilizers.

*Astragalus mongolicus* Bunge (*A. mongolicus*) is a perennial leguminous herb, growing mainly in Shanxi Province, Gansu Province, and Inner Mongolia Province, amongst other places in China [18]. *A. mongolicus* is often used as a traditional Chinese medicine because of its functions: Tonifying Qi and strengthening the exterior, tonifying spleen and Qi, diuresis, and detumescence [19]. At the same time, it is also widely used in various clinical disciplines as it is efficacious in treating various conditions and disorders. The main secondary metabolites of *A. mongolicus* include flavonoids, saponins, and polysaccharides, all of which have a wide range of pharmacological effects. In recent years, the market consumption of *A. mongolicus* has increased, resulting in the gradual depletion of wild *A. mongolicus* resources [20]. Thus, *A. mongolicus* for commercial use is largely artificially cultivated to meet the market demand. The application of chemical fertilizer is generally used in Gansu and Shanxi, the main cultivation areas, to improve the yields, which not only significantly reduces the quality of *A. mongolicus* but also has adverse effects on the environment. In addition, the nitrogen-fixing bacteria isolated from the other plants bind poorly to *A. mongolicus*. Therefore, the application of special organic nitrogen-fixing bacteria fertilizer for ecological planting is an appropriate choice to improve the quality of *A. mongolicus* and implement environmental protection policy.

In the previous study, we isolated two strains of highly efficient rhizobium of *A. mongolicus* from its authentic producing area. The experiments show that the rhizobium has a strong symbiotic ability with *A. mongolicus* and high nitrogen fixation efficiency, which can promote the growth of *A. mongolicus,* and has been applied in field production [21, 22]. Meanwhile, efficient non-symbiotic nitrogen fixing bacteria also need to be screened, and then combined with symbiotic rhizobia to form a multi-base combined PGPR bacterial agent, thus promoting the nitrogen utilization of Astragalus more effectively. In this study, we aimed to screen combined nitrogen-fixing bacteria in the rhizosphere of *A. mongolicus* for plant growth promotion.

In this study, nitrogen-fixing bacteria were isolated from rhizospheres in the *A. mongolicus* cultivation area, and experiments were conducted to identify high-efficiency nitrogen-fixing bacteria of *A. mongolicus*. Finally, antagonistic symbiosis experiments were carried out on the isolated high-efficiency nitrogen-fixing bacteria. The symbiotic strains were prepared into multi-species nitrogen-fixing bacteria agents and used in field experiments. The overall objective was to contribute to developing a specific bacterial fertilizer for *A. mongolicus* to reduce the problems caused by the excessive use of chemical fertilizers.

## Materials and methods

### Sample collection

In this study, two-year-old *A. mongolicus* plants were selected from the major production area, experimental base of Longxi County, Gansu Province, Jingle County, and Hunyuan County, Shanxi Province. The root tissue and rhizosphere soil were collected in sealed bags, numbered, and stored at a low temperature (4 ℃) for later use.

### Isolation and purification of potential nitrogen-fixing bacteria

The root tissues were surface-sterilized (washed in 95% ethanol for 1 min, 3% NaOCl for 5 min, a 30s wash in 99% ethanol, and rinsed with sterile water), crushed with tweezers, streaked on Ashby nitrogen-free medium, and cultured at 28 ℃ for 5 d to select a typical single colony. The isolated strains were purified and stored at -80 ℃ for later use.

The fresh rhizosphere soil was diluted with sterile water for 10^4^, 10^5^ and 10^6^ times respectively to make bacterial suspension. One hundred microliter aliquots of the diluted material were then spread separately on Ashby nitrogen-free medium and cultured at 28 ℃ for 5 d, after which a typical single colony was selected from each inoculum, purified, and stored at -80 ℃ for later use.

### Determination of the nitrogen fixation efficiency of strains

The nitrogen fixation efficiency of the stain was measured by the acetylene reduction activity (ARA) assay method. Each strain to be tested was inoculated into 100 mL modified Dobereiner medium and cultured with shaking at 28 ℃ and 120 rpm for 3 days. The ARA assay was conducted following the procedure described in a previous study [23].

The strains with high nitrogenase activity were preliminarily selected and inoculated into 100 mL Döbereiner nitrogen-free liquid medium respectively and cultured with shaking at 28 °C and 120 rpm for 7 d. The nitrogen content of the supernatant was determined by the micro-Kjeldahl method [24].

### Strain identification

#### Physiological and biochemical characteristics of strains

Most physiological and biochemical tests, including citrate hydrolysis, the bromothymol blue (BTB) reaction, ester hydrolysis, Voges-Proskauer (VP) reaction, litmus acidification reaction, and growth at 41 ℃ with 2% sodium chloride, were observed using the methods described by Dong XZ and Cai MY [25]. Starch hydrolysis and 3-ketolactose hydrolysis were measured by the method described by Smibert and Krieg [26].

#### Molecular identification of strains

The strains were inoculated in the enrichment medium and cultured with shaking at 28 ℃ and 180 rpm for 2 days. Then, 3 ml of the culture medium was centrifuged at 10 000 rpm for 3 min to collect the bacteria. Bacterial genomic DNA was extracted according to the instructions of the extraction kit(RTG2401-01 Real-Times Biotechnology Co., Ltd., Beijing, China). The genomic DNA of the tested strain was extracted according to the manual of the reference kit and used for PCR amplification with the bacterial 16S rRNA universal primer 27F (5’-AGAGTTTGATCCTGGCTCAG-3’) /1492R (5’-TACGACTTAACCCCAATCGC-3’). The reaction system and conditions used are shown in Table 1. The PCR products were detected by 1% agarose gel electrophoresis and sequenced. The sequencing results were compared with bacterial nucleotide sequences using NCBI BLAST. The phylogenetic tree was constructed with Mega-X software to determine the genetic relationships between the strains [27].

**Table 1:** PCR reaction system and conditions of 16S rRNA

Reaction system	2 × mix 20 µL, Primer27F 1.5 µL, Primer1492R 1.5 µL, ddH_2_O 15 µL, Template gDNA 2 µL			
Reaction process	Predenaturation	Transsexual	Refolding	Extend
Temperature Reflex	95 ℃	95 ℃	52 ℃	72 ℃
Reaction time	3 min	30 s	30 s	7 min

#### Scanning electron microscope sample preparation

The strains were inoculated in the enrichment medium and cultured with shaking at 28 ℃ and 180 rpm for 2 days. Next, 2 ml of the culture medium was centrifuged at 10 000 rpm for 3 min, and the precipitate was fixed in 2 mL of glutaraldehyde(Solarbio P1126 2.5% glutaraldehyde) fixative solution at 4 ℃ for at least 2 h. The fixed samples were then rinsed with phosphate buffer two to three times and dehydrated in an ethanol gradient of 50%, 70%, 90%, and 100%. After dehydration, the samples were immersed in ethanol-tert-butanol solution for 20 min and replaced in 100% tert-butanol solution twice for 20 min each. The samples were then freeze-dried, sputtered with an ion-sputtering apparatus, and observed and photographed with a scanning electron microscope.

### Antagonistic test of strains

The two strains were inoculated on YEM solid plate medium vertically and horizontally, cultured in an incubator at 28 ℃ for 20 min, followed by culturing upside down for 3 days. Bacterial growth at the vertical and horizontal lines intersection was monitored daily. The presence of growth inhibition at the intersection indicates antagonism and that the two strains would not be suitable for mixed culture, while an absence of growth inhibition at the intersection implies no antagonism and suitability for mixed culture.

### Strain inoculation test

The strains were inoculated into YEM liquid medium and cultured with shaking at 28 ℃ and 180 rpm for 72 h to obtain the bacterial liquid. At this point, the concentration of bacteria in the medium, measured by spectrophotometry, was greater than 1×1010 CFU.mL-1. The bacterial solution was diluted 1:100 with distilled water, and *A. mongolicus* seeds were soaked in the bacterial solution (concentration about 108 CFU.mL-1) and then were sown in a sterile seedling substrate, with distilled water treatment as control. The *A. mongolicus* seedlings were cultured at 26 ℃ and 12 000 lx light intensity with daily light, watered with 2 ml of water daily, transplanted after 15 d, and then watered with 7 ml of water daily. After 30 d, the whole seedlings were dug out, and samples in each group were randomly chosen to determine the biomass and the nitrogen, phosphorus, and potassium contents. The biomass indicators included plant height, root length, and dry weight, both above- and below-ground. The samples were dried and digested to determine the total nitrogen, total phosphorus, and total potassium content using the Kjeldahl method [28], the molybdenum antimony colorimetric method [29], and the flame photometer method, respectively [30].

### Preparation of bacterial fertilizer and field inoculation test

The isolated nitrogen-fixing bacteria were individually inoculated into YEM liquid medium and cultured with shaking at 28 ℃ and 180 rpm for 72 h to prepare a mixed bacterial stock solution (the bacterial content was approximately 1×1010 CFU.mL-1). The bacterial stock solution was diluted 100 times into liquid bacterial fertilizer (bacterial content was approximately 108 CFU.mL-1). The field experiment was carried out in May 2019 in the main production fields of *A. mongolicus*, Hunyuan County, Shanxi Province (39◦516 N, 113◦643 E, altitude: 1238 m). The new *A. mongolicus* seed from Hunyuan County was used as the test material. The seeds were first soaked with the bacterial solution for an hour and then sown in the experimental field. Distilled water was used as the control treatment. The area of each plot was 167 m2, and the seeding rate was 1.05 kg. Ditches 20 cm wide were made between the rows of *A. mongolicus*, and the inoculated seeds were spread evenly in the ditch to cover the soil at the base. Each plot test contained three replicates. Random sampling was performed at the June, July, August, September, and October stages of *A. mongolicus*.

### Determination of A. mongolicus biomass and active component content

The soil was cleaned from the surface of the plants, using absorbent paper to absorb the water. After measuring the height and root length of the plants with a ruler, the plants were dried to a constant weight at 70 ℃, and the aerial parts and roots were weighed.

The dry roots were ground and sieved through a 55-mesh sieve. One g of the coarsely ground powder was added to 5% ethanol with a material-to-liquid ratio of 1:20 and flash-extracted for 1 min at 120 V and 4 ℃. After centrifugation at 5000 rpm for 10 min, the supernatant was rotary-evaporated at 55 ℃ and 60 rpm to yield 2 mL of concentrated solution. The concentrated solution was precipitated overnight (12 h) in four times its volume of anhydrous ethanol and centrifuged at 8000 rpm and 4 ℃ for 5 min. Finally, the resultant precipitated and dried polysaccharide was used to determine the total polysaccharide content by the phenol-sulfuric acid method [31].

After being ground and sieved through a 55-mesh sieve, 1 g of a coarse powder was added to 85% ethanol in a material-to-liquid ratio of 1:20, flash-extracted at 120 V for 1 minute, and centrifuged at 4 ℃ and 5000 rpm for 10 minutes. The supernatant was evaporated at 55 ℃ and 60 rpm to 2 mL of concentrated solution. The concentrated solution was diluted to 10 mL with 30% ethanol and extracted three times with saturated ethyl acetate with water to recover the ethyl acetate layer. The residue was evaporated to dryness in a water bath to obtain the flavonoid sample. The water layer was then extracted with water-saturated n-butanol three times to recover the n-butanol. The residue was then evaporated to dryness in a water bath to obtain a saponin sample. The total flavonoid content was determined by spectrophotometry, and the total saponins were determined by the vanillin-concentrated sulfuric acid method [32].

### High-throughput sequencing and analysis of 16S rRNA

Soil samples before sowing (CK0) and rhizosphere soil samples at 90 days after field experiment conduction (CK: control and T: bacterial fertilizer treatment) were collected in triplicate for bacterial community analysis.

DNA of soil samples was extracted with an Omega E.Z.N.A soil DNA extraction kit. The V3-V4 region of bacterial 16S rRNA genes were amplified using the universal primer pair F338/R806 (F338, 5′-ACTCCTACGGGAGGCAGCAG-3′; R806, 5′-GGACTACHVGGGTWTCTAAT-3′). PCR reactions were performed using TransStart Fastpfu DNA Polymerase 20µl reaction system under the following condition: 95 ℃ for 2 min, followed by 35 cycles at 95 ℃ for 30 s, annealing at 55 ℃ for 1 min, extension at 72 ℃ for 1 min, and a final extension at 72 ℃ for 10 min. Purified amplicons were paired-end sequenced on an Illumina MiSeq PE300 platform (Illumina, Inc., Santiago, CA, USA) at Majorbio Bio-pharm Technology Co., Ltd (Shanghai, China).

Illumina raw reads were processed by the QIIME2 pipeline [33]. The paired-end sequences were merged and renamed according to the sample barcode using Vsearch package. The barcode and primer sequences were trimmed, and then quality filtering, dereplication, clustering, and chimera-removing were conducted in the QIIME2 pipeline. The cleaned sequences were clustered into Operational taxonomic units (OTUs) at a 97% similarity level. Taxonomy annotation of each OTU representative sequence was performed using the blast algorithm and the greengenes database (version 13.5) [34].

### Statistical analysis

Average values and standard deviations were computed according to the experimental data. Using SPSS 25.0 statistical package, a one-way analysis of variance (ANOVA) with Duncan's test was conducted on the data, and a *p*-value < 0.05 was considered a significant level.

## Results

### Isolation of nitrogen-fixing bacteria from A. mongolicus

Using YEM solid medium and Ashby nitrogen-free medium, 27 and 59 strains with potential nitrogen fixation ability were isolated from root tissue and rhizosphere soil, respectively. Of these, 30 strains were isolated from Longxi County, Gansu Province, 25 from Jingle County, Shanxi Province, and 31 from Hunyuan County, Shanxi Province.

### Screening for nitrogen-fixing efficiency of stains

The results of nitrogen fixation experiments showed that among the 86 strains, 34 strains were able to fix nitrogen (Table 2). Among them, 14 strains were isolated from root tissue, and 20 were isolated from soil. Among all the tested strains, 15 strains from Longxi had a nitrogenase activity of 0.45-37.45 nmol C2H4/(mL·h), nine strains from Jingle had a nitrogenase activity of 0.14-67.45 nmol C2H4/(mL·h), and 10 strains from Hunyuan had a nitrogenase activity of 14.32-31.45 nmol C2H4/(mL·h). Among the 34 strains of nitrogen-fixing bacteria, only two strains had a nitrogenase activity higher than 50 nmol C2H4/(mL·h), namely J1 and J2 (collected from Jingle County). The strain J1 (isolated from root tissue) had the highest nitrogenase activity of 67.45 nmol C2H4/(mL·h).

**Table 2:** Nitrogenase activity of 34 stains of potential nitrogen-fixing bacteria

Source	Sampling site	Strains	Nitrogen fixation Efficiency (nmol C_2_H_4_/(mL·h))	Source	Sampling site	Strains	Nitrogen fixation Efficiency (nmol C_2_H_4_/(mL·h))
Nodule	Longxi County	G4	37.45	Soil	Longxi County	G18	11.1
Nodule	Longxi County	G7	16.75	Soil	Longxi County	G19	20.35
Nodule	Longxi County	G8	15.22	Soil	Longxi County	G22	2.83
Nodule	Longxi County	G9	5.75	Soil	Longxi County	G23	19.55
Root	Longxi County	G13	0.45	Soil	Longxi County	G27	6.21
Root	Longxi County	G16	11.83	Soil	Jingle County	J2	59.35
Root	Longxi County	G21	10.85	Soil	Jingle County	J15	0.14
Root	Jingle County	J1	67.45	Soil	Jingle County	J17	6.05
Root	Jingle County	J4	24.05	Soil	Jingle County	J22	3.11
Root	Jingle County	J8	19.35	Soil	Hunyuan County	H3	25.75
Root	Jingle County	J14	11.61	Soil	Hunyuan County	H4	14.32
Root	Jingle County	J25	12.15	Soil	Hunyuan County	H6	16.65
Root	Hunyuan County	H2	22.65	Soil	Hunyuan County	H13	31.45
Root	Hunyuan County	H7	27.65	Soil	Hunyuan County	H15	21.84
Soil	Longxi County	G2	3.15	Soil	Hunyuan County	H16	26.55
Soil	Longxi County	G3	13.15	Soil	Hunyuan County	H20	27.05
Soil	Longxi County	G6	17.73	Soil	Hunyuan County	H22	20.34

The strains G4, J1, H7 (isolated from root tissue) and strains J2, H3, H13, H16, H20 (isolated from rhizosphere soil) with good growth characteristics and nitrogen fixation efficiency were selected for further analysis (Table 3).

**Table 3:** Nitrogen fixation capacity of strains

Strain	G4	J1	H7	J2	H3	H13	H16	H20
Nitrogen fixation (mg/L)	12.94 ± 0.38	19.65 ± 0.52	6.57 ± 0.37	16.77 ± 0.87	6.03 ± 0.23	7.24 ± 0.32	5.34 ± 0.28	8.11 ± 0.22

### Identification of stains

#### Phenotypic characteristics of strains

Nitrogen fixation for solid culture, and the phenotypic characteristics of the individual clones of each strain were observed. The results (Table 4) showed that although all colonies were white and opaque and the colony surfaces were smooth and moist, no obvious hyphae were visible. The colony characteristics of the eight tested strains varied between species, with the G4 and H3 colonies flowing easily, while the J1 and H7 colonies were raised, flat, and did not flow easily, and the J2, H13, H16, and H20 colonies were raised and flowed easily.

**Table 4:** Phenotypic characteristics of the eight tested strains

Strain	Color	Transparency	The edge	Surface condition	Uplift degree
G4	White	Opaque	Smooth and tidy	Moist, not easy to flow	Raised
J1	White	Opaque	Smooth and tidy	Moist, not easy to flow	Flat
J2	White	Opaque	Smooth and tidy	Moist, easy to flow	Raised
H3	White	Opaque	Smooth and tidy	Moist, not easy to flow	Raised
H7	White	Opaque	Smooth and tidy	Moist, not easy to flow	Flat
H13	White	Opaque	Smooth and tidy	Moist, easy to flow	Raised
H16	White	Opaque	Smooth and tidy	Moist, easy to flow	Raised
H20	White	Opaque	Smooth and tidy	Moist, easy to flow	Raised

#### Scanning electron micrographs of strains

**Fig. 1:**
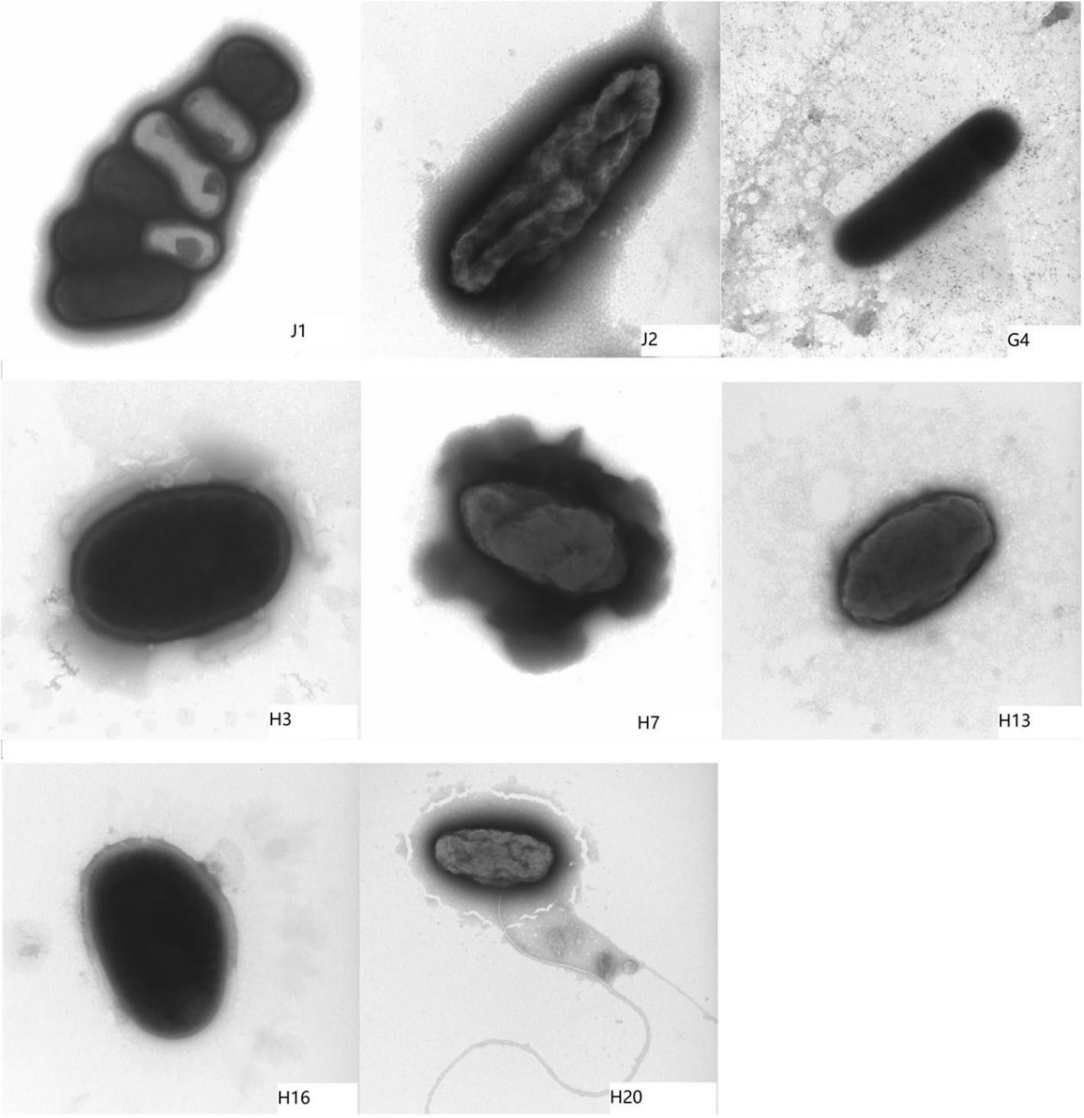
Electron micrograph of 8 tested strains (J1, J2, G4, H3, H7, H13, H16 and H20)

The results of the scanning electron microscopy (Fig. 1) showed that eight tested strains were all rod-shaped with inconspicuous hyphae. Among them, in J1, the bacilli were clustered with a size range of 0.1-0.15×0.5-0.75 μm, while J2 and G4 were composed of single bacilli in the size range of 0.1-0.15 ×0.75-8 μm, and H3, H7, H13, H16, H20 were visible as cocci in clusters with diameters of 0.2-0.3 μm.

#### Physiological and biochemical identification of strains

Physiological and biochemical tests were carried out on the eight selected strains. The results are shown in Table 5. The results showed that the eight experimental strains lacked esterase, had a low rate of utilization of peptone in beef extract, could not use lactose in milk, could not grow in BTB medium, and did not belong to the *Agrobacterium* genus. All the experimental strains could degrade citric acid, have good heat resistance and salt tolerance, and use citrate. All strains except H7, H13, and H20 contained contact enzymes. All strains except H16 contained amylase. Strains J2, H3, and H16V-P had negative reactions, and strains G4, J1, H7, H13, and H20V-P had positive reactions.

**Table 5:** Physiological and biochemical identification of 8 strains of nitrogen-fixing bacteria

Strain	Source	Acidogenesis of litmus milk	Beef peptone medium	3-keto lactose reaction	Catalase test	V-P	41℃	2%NaCl	Esterase reaction	BTB	Amylase reaction	Citrate test
G4	Nodule	-	-	-	+	+	+	+	-	-	+	+
J1	Nodule	-	-	-	+	+	+	+	-	-	+	+
H7	Nodule	-	-	-	-	+	+	+	-	-	+	+
J2	soil	-	-	-	-	+	+	+	-	-	+	+
H3	soil	-	-	-	+	-	+	+	-	-	+	+
H13	soil	-	-	-	-	+	+	+	-	-	+	+
H16	soil	-	-	-	+	-	+	+	-	-	-	+
H20	soil	-	-	-	-	+	+	+	-	-	+	+

" + " means a positive reaction, and "-" means a negative reaction

#### Molecular identification of strains

**Fig. 2:**
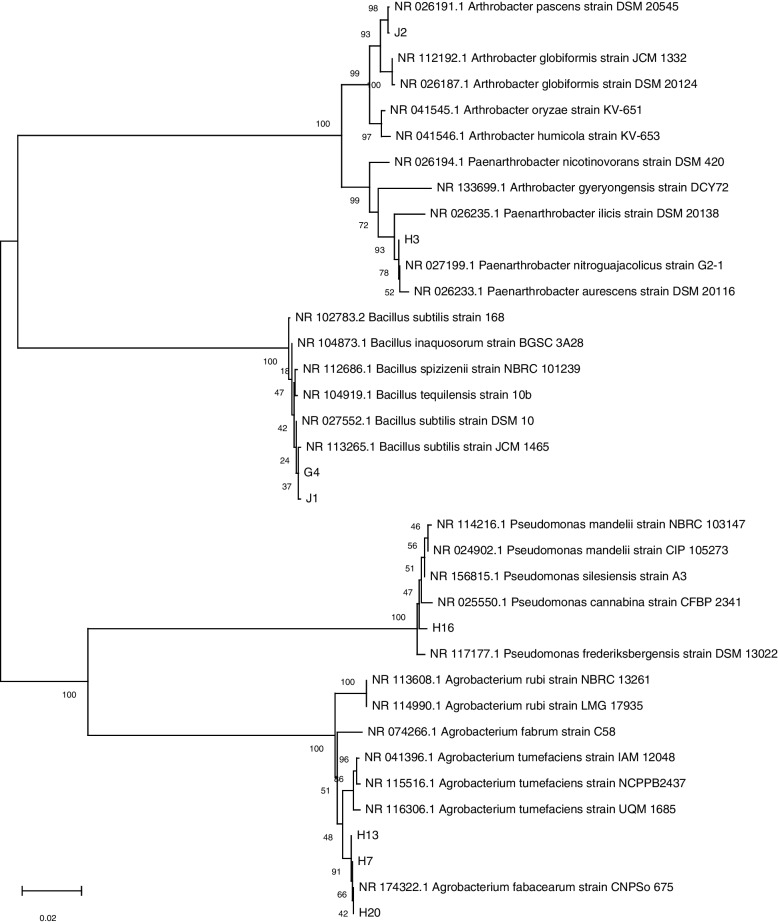
Molecular identification of 8 tested strains of nitrogen-fixing bacteria

The 16S rDNA sequences of the screened bacteria were compared using NCBI BLAST, and the phylogenetic tree was constructed. As shown in Fig. 2, all tested strains were phylogenetically distributed with five genera: *Agrobacterium, Pseudomonas, Bacillus, Paenarthrobacter*, and *Arthrobacter*. The 16S rRNA sequence analysis showed that *Agrobacterium* included three genotypes. The H7, H13, and H20 strains were the most similar to the reference strain *Agrobacterium fabacearum* CNPSo 675, with a similarity of 99.8%. *Pseudomonas* included one genotype. The similarity between strain H16 and *Pseudomonas_silesiensis A3* was 99.3%. *Bacillus* contained two genotypes. Strains J1 and G4 were the closest to the reference strain *Bacillus subtilis JCM* 1465, and the similarity was 99.8%. *Paenarthrobacter* included one genotype. Strain H3 was identical (100% similarity) to *Paenarthrobacter nitroguajacolicus G2-1*. *Arthrobacter* included one genotype, and the similarity between strain J2 and *Arthrobacter pascens DSM 20545* was 99.9%.

### Effects of the different strains on A. mongolicus

#### Effects of the strains on the biomass of A. mongolicus

**Fig. 3:**
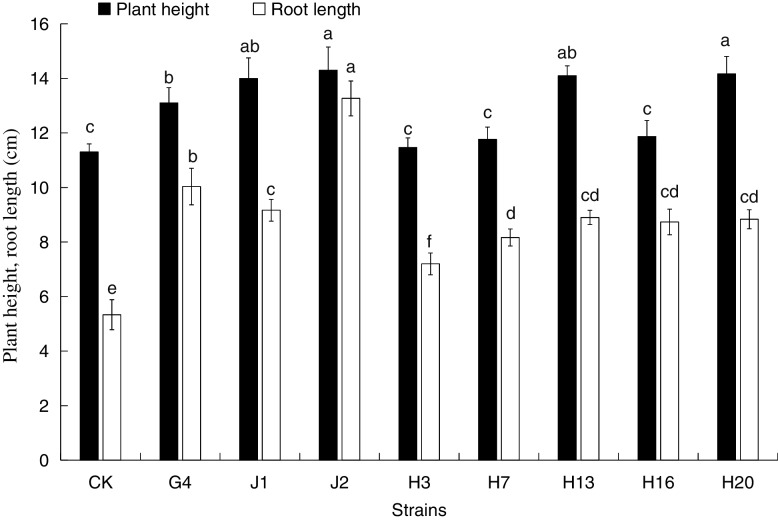
Effect of the tested strain on the height and root length of *A. mongolicus*. Different letters indicate the significant difference between treatments according to ANOVA with Duncan's test (*p* < 0.05)

**Fig. 4:**
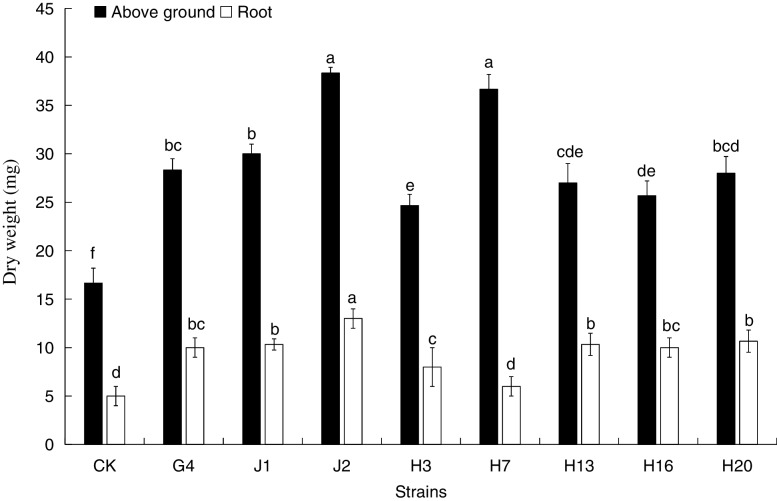
Effect of test strain on the dry weight of *A. mongolicus*. Different letters indicate the significant difference between treatments according to ANOVA with Duncan's test (*p* < 0.05)

According to the results of the inoculation experiment shown in Figs. 3 and 4, compared with the control group, the inoculated seedlings showed varying degrees of changes in plant height, root length, above-ground dry weight, and root dry weight. Among them, strains J1, J2, G4, H13, and H20 significantly increased the *A. mongolicus* plant height. The growth effects of J1, J2, and G4 on the plant roots were obvious. The plants' dry weights of aerial parts were significantly increased by the J1, J2, G4, H7, and H20 strains, while J1, J2, G4, H13, H16, and H20 significantly promoted the root dry weight. Based on the above data, we concluded that the growth of *A. mongolicus* was most significantly promoted by the J1, J2, and G4 strains.

#### Effects of the strains on nitrogen, phosphorus, and potassium contents of A. mongolicus

**Fig. 5:**
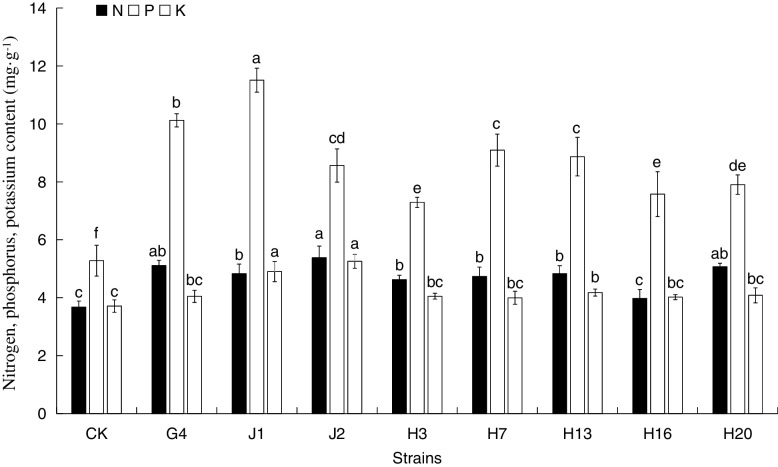
Effects of the tested strain on nitrogen, phosphorus, and potassium contents of *A. mongolicus*. Different letters indicate the significant difference between treatments according to ANOVA with Duncan's test (*p* < 0.05)

The nitrogen, phosphorus, and potassium contents of *A. mongolicus* seedlings after inoculation with different strains are shown in Fig. 5. It can be seen from the results that different strains had different effects on these indicators. In the natural growth state (CK), the nitrogen content of the seedlings was 3.68%, the phosphorus content was 5.28%, and the potassium content was 3.71%. Except for strain H16, the treatment indices of the other strains were significantly higher than those of the control. Compared with the control, the seedlings' nitrogen, phosphorus, and potassium contents were significantly increased by 8.07%-46.32%, 38.07%-117.96%, and 7.74%-41.66%, respectively. Strain J2 had the most obvious effect on nitrogen content, with a significant increase of 46.32%, followed by G4, which increased by 38.86%; The effect of strain J2 on the potassium content was the most significant, increasing by 41.66%, followed by J1 which increased by 32.08%. The phosphorus content of seedlings inoculated with strain J1 increased the most by 117.96%, followed by G4 with 91.67%. The growth-promoting effect of the strains was consistent with the increases in nitrogen, phosphorus, and potassium contents. It could be seen that inoculation of the tested strains could significantly promote not only the growth of *A. mongolicus* seedlings but also the absorption and utilization of nitrogen, phosphorus, and potassium. Moreover, the strains could effectively promote the absorption of phosphorus by the plant. The increase in the phosphorus content indicates an increase in the ATP content in the plants, which provides a material basis for both rapid growth and metabolism.

### Antagonism of strains

**Fig.6:**
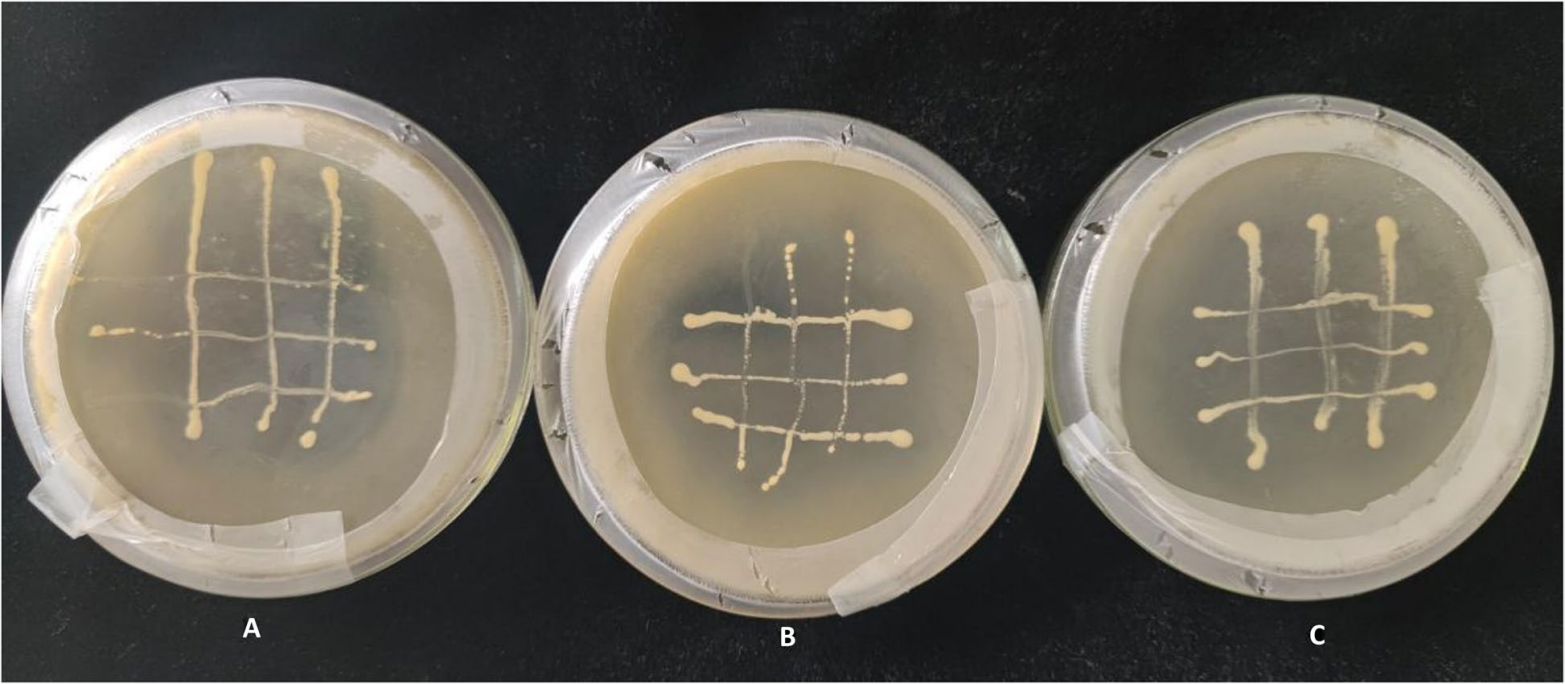
Experimental results of paired antagonism test of strains J1, J2, and G4 (A: J1 and J2, B: J1 and G4, C: J2 and G4)

Experiments to test antagonism were performed with the three J1, J2, and G4. It can be seen from Fig. 6 that the three bacteria did not affect each other's growth at the junction, indicating that the three bacteria are not antagonistic to each other and can thus be co-cultured.

### Growth-promoting Effects of mixed bacterial fertilizer on A. mongolicus

#### Effects of mixed microbial agents on the biomass of A. mongolicus

**Fig. 7:**
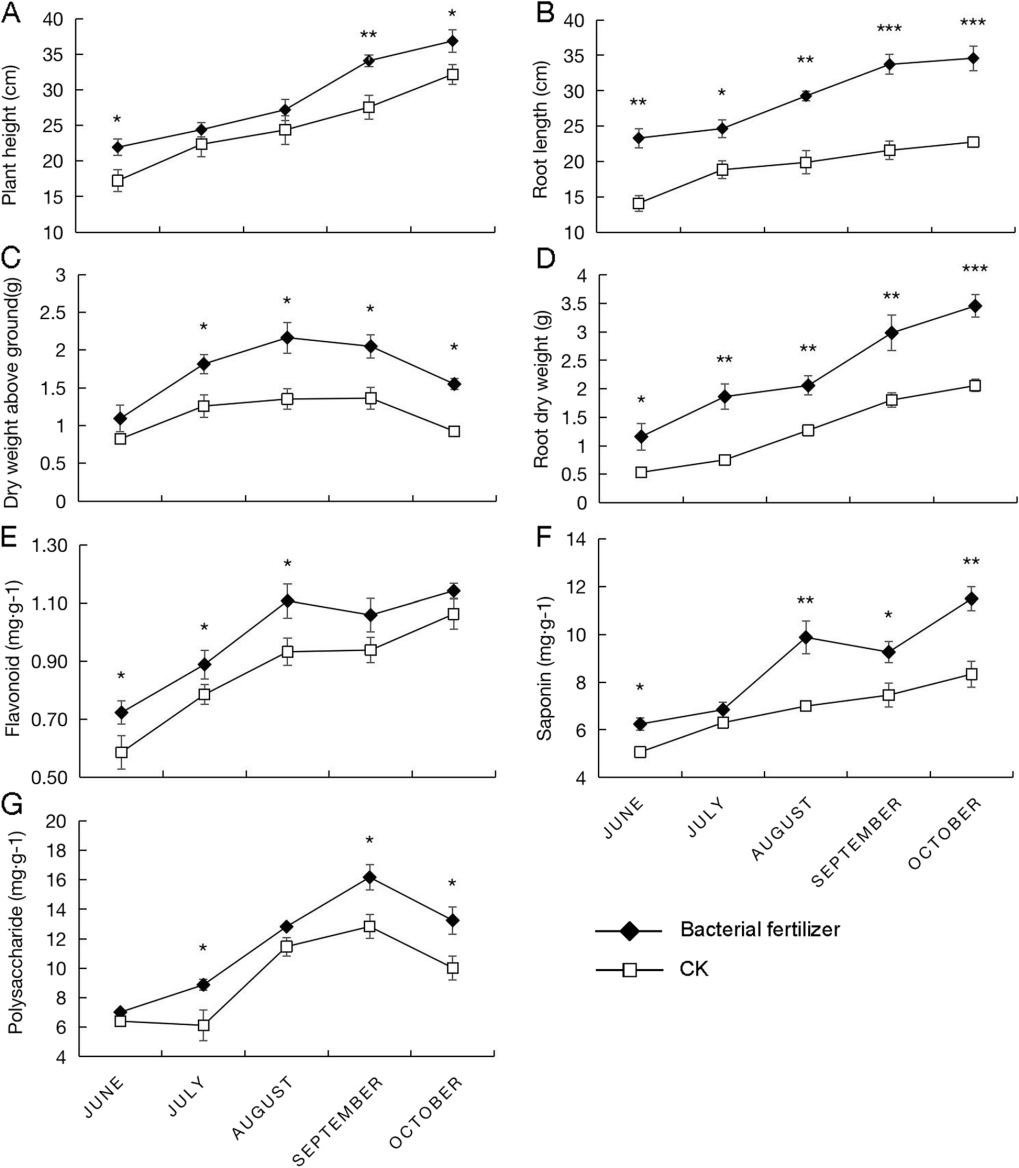
Effects of bacterial fertilizer on biomass and effective components of *A. mongolicus*. **A** is the effect on the plant height, (**B**) is the effect on the root length, (**C**) is the effect on the dry weight of the aerial part of *A. mongolicus*, (**D**) is the effect on the dry weight of the root, E is the effect on the content of astragalus flavone, (**F**) is the effect on the content of astragaloside, (**G**) is the effect on the content of astragalus polysaccharide. *, **, and *** indicate significant differences between bacterial fertilization and control at *p* < 0.05, *p* < 0.01, and *p* < 0.001, respectively

We selected the better-performing J1, J2, and G4 strains to make a mixed bacterial liquid used to dress the *A. mongolicus* seeds in an artificial climate chamber, after which they were sown in the field. The plant height, root length, and dry weights of the aerial parts and roots of the plants at different growth stages were measured and compared with the plants seeded with distilled water. As shown in Fig. 7 (A, B, C, D), the bacterial fertilizer significantly enhanced the plant height, root length, and dry weights of the above-ground parts and roots of the plants during the different growth periods. Compared with the control group, there are significant differences in plant height in June, July, September, and October. The difference in September is extremely significant. Plant height increased the most in September, an increase of 23.66% compared to the control. Root growth occurred in June, July, and August, and the difference between September and October is extremely significant. The root length increased the most in September, an increase of 56.16% compared to the control. The difference in root dry weight is significant between June and July, August and September, and the difference in October was extremely significant. The root dry weight increased the most in October, 68.06%, compared to the control; the dry leaf weight was extremely different in July, August, and October. The dry weight of the above-ground part increased the most in August, an increase of 60.18% compared to the control.

#### Effects of mixed microbial agents on the active components of A. mongolicus

As shown in Fig. 7 (E, F, G), the bacterial fertilizer could significantly promote the accumulation of flavonoids, saponins, and polysaccharides, which are the main active components of *A. mongolicus*. The flavonoid content differed significantly in June, July, and August. The flavonoid content reached the largest difference in June, which increased by 23.41% compared to the control. The saponin content significantly differed in June, August, September, and October. The difference is extremely significant in October. The difference in saponins content reached the largest in September, an increase of 41.21% compared to the control; the polysaccharide content was significantly different in July, September, and October. The polysaccharide content difference reached the largest in July, an increase of 44.87% compared to the control.

### Effects of mixed microbial agents on soil bacterial communities of A. mongolicus

**Fig.8:**
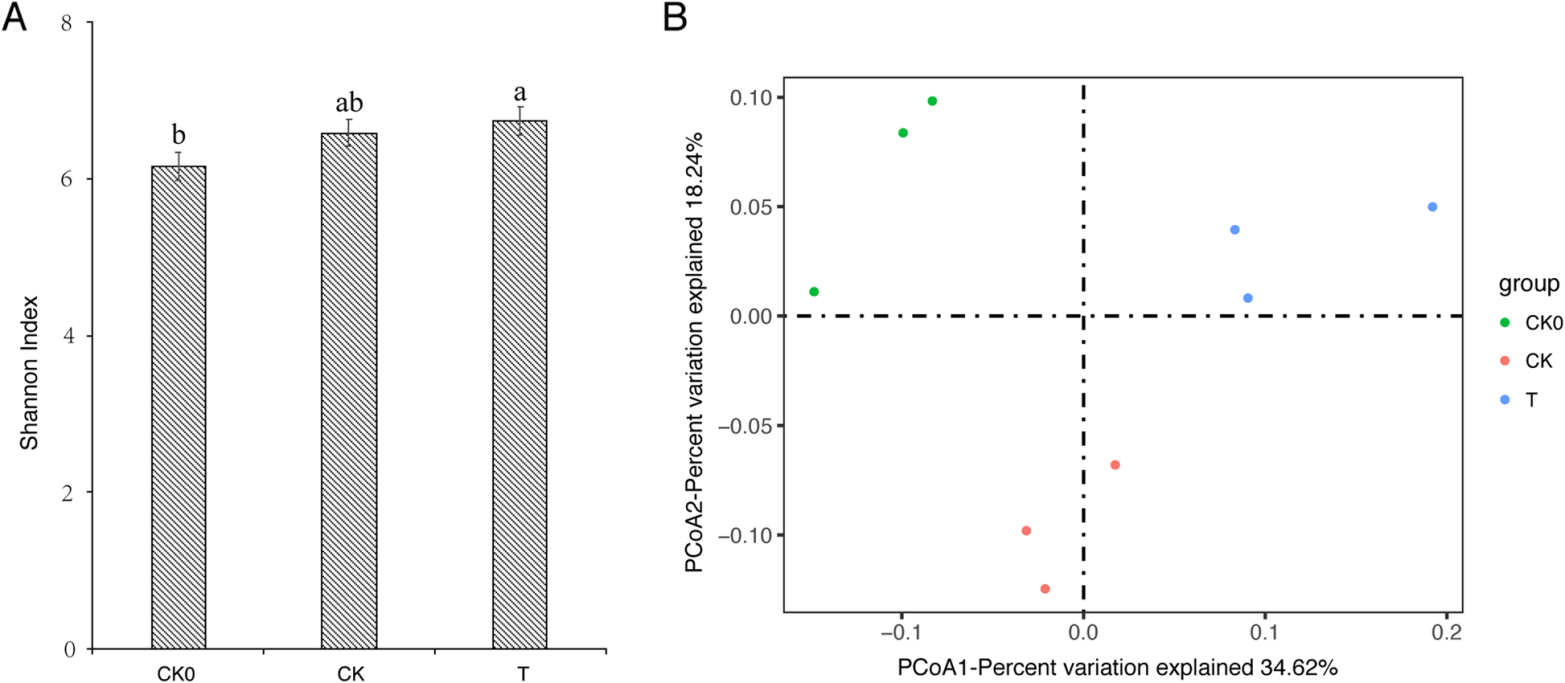
Effect of bacterial fertilizer on the bacterial diversity and community structure. **A** Shannon index of the bacterial community, (**B**): principal coordinate analysis of bacterial communities in different groups. CK0, soil samples before sowing. CK, rhizosphere soil samples at 90 days after planting without fertilizer, T, rhizosphere soil samples at 90 days after planting with bacterial fertilizer treatment. Different letters indicate the significant difference between treatments according to ANOVA with Duncan's test (*p* < 0.05)

The bacterial community of rhizosphere soil samples was analyzed using the Illumina MiSeq PE300 platform and QIIME2 pipeline. A total of 109821 raw reads were sequenced and finally clustered into 2655 OTUs for bacteria after removing singletons. Shannon index showed that the diversity of the bacteria community was significantly improved after planting *A. mongolicus* (*p* < 0.05). However, diversity indices between experimental and control groups showed no significant differences in bacterial diversity (*p* > 0.05) (Fig. 8A). Meanwhile, PCoA analyses were performed to evaluate the structural similarities between bacterial communities in all groups. The result indicated that the bacterial communities in the treatment and control groups had a significant difference (Fig. 8B).

**Fig.9:**
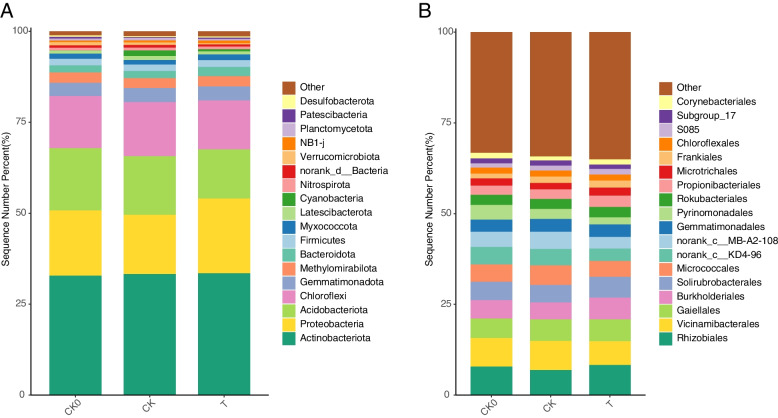
Relative abundance of bacteria at phyla (**A**) and order (**B**) levels of different groups. CK0: soil samples before sowing. CK: rhizosphere soil samples at 90 days after planting without fertilizer, T: rhizosphere soil samples at 90 days after planting with bacterial fertilizer treatment

The dominant bacterial phyla identified in the special manure group were Actinobacteriota, Proteobacteria, Acidobacteriota, Chloroflexi, Gemmatimonadota, Methylomirabilota, and Bacteroidota. In the treatment group, proteobacteria increased from the relative abundance of 17.88% before sowing (CK0) to 20.48% (T) and relatively decreased to 16.27% in the control group (CK). Compared with that before sowing, the abundance of *Acidobacteriota* decreased in the treatment group (13.53%) and the control group (16.11%), and the decrease was more significant in the treatment group. The abundance of *Actinobacteriota* was the highest in all groups and remained stable (average: 33.21%) (Fig. 9A). At the order level, Rhizobiales, Vicinamibacterales, Gaiellales, Burkholderiales, Solirubrobacterales and Micrococcales were the dominant bacterial order. Rhizobiales, Burkholderiales and Solirubrobacterales were increased in the treatment group and decreased in the control group. Correspondingly, the trend of Vicinamibacterales and Micrococcales is the opposite (Fig. 9B).

**Fig.10:**
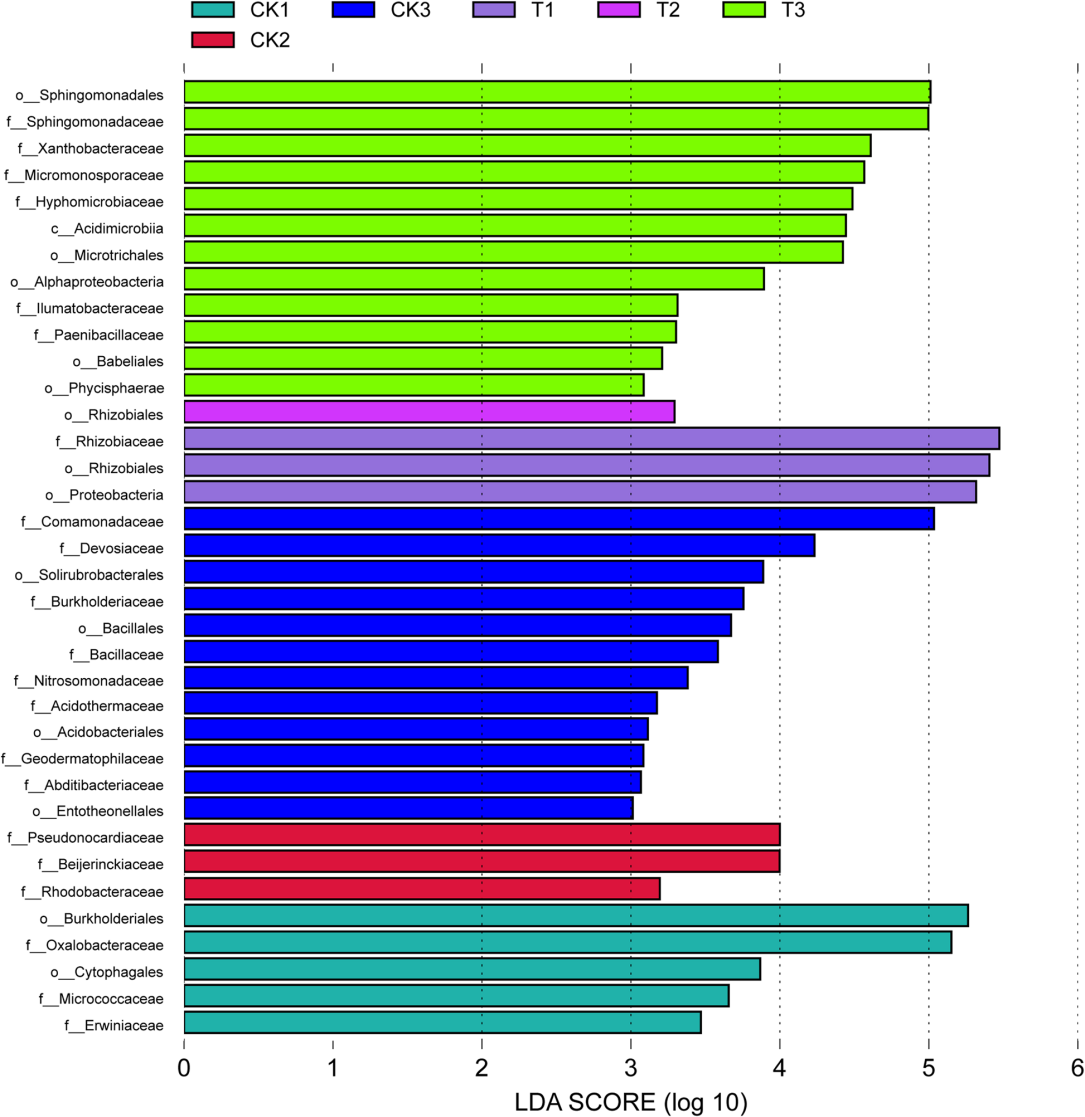
Lefsee analysis of significantly different in rhizosphere soil bacteria at family leval. CK: rhizosphere soil samples at 90 days after planting without fertilizer, T: rhizosphere soil samples at 90 days after planting with bacterial fertilizer treatment

Lefsee method was used to analyze the statistical differential abundance of rhizosphere bacterial community among groups. The results showed that Rhizobiaceae, Sphingomonadaceae, Xanthobacteraceae, Micromonosporaceae and Hyphomicrobiaceae showed the largest difference in treatment group. Meanwhile, Oxalobacteraceae, Comamonadaceae and Devosiaceae had the largest difference in control group (Fig. 10).

## Discussion

In this study, 86 strains of nitrogen-fixing bacteria were isolated from the root tissue and rhizosphere of *A. mongolicus* in Jingle County, Shanxi Province, Hunyuan County, Shanxi Province, and Longxi County, Gansu Province. Previous studies have shown that it is difficult to isolate high-efficiency nitrogen-fixing bacteria from the rhizosphere of leguminous plants such as *Camellia oleifera* [17], and the numbers of high-efficiency nitrogen-fixing bacteria isolated in this study were also less. It is speculated that the population numbers of nitrogen-fixing bacteria in the rhizosphere of *A. mongolicus* are relatively small, suggesting limited competition with other microorganisms.

Eight species of bacteria isolated from the *A. mongolicus* rhizosphere were identified by 16S rRNA sequencing. These eight species were distributed over five genera, namely, *Agrobacterium* [35], *Pseudomonas* [36], *Bacillus* [37], *Paenarthrobacter* [38], and *Arthrobacter* [39] with nitrogen-fixation ability. Some strains that can fix nitrogen also have other functions. For example, *Agrobacterium* is an important vector in genetic engineering, *Paenarthrobacter* [40] and *Arthrobacter* [41] are used for treating industrial wastewater and soil pollution, and for promoting plant growth, *Bacillus* is an important industrial microorganism used in cellulose degradation [42], while *Pseudomonas* is one of the most studied bacteria for use in biocontrol applications [43]. The results further indicated that regional differences influenced microbial species and that strains isolated from the same host in different regions may have relatively distant genetic relationships. The rhizosphere microbial community structure of the same host in the same area is also very complex.

Many studies have described the screening and application of nitrogen-fixing bacteria in legumes, including soybean [44], chickpea [45], and alfalfa [46]. Studies have shown that nitrogen-fixing bacteria can significantly increase yields and improve soil fertility and soil microbial community structure [47], as well as significantly impacting the protein content [48]. Studies have shown that nitrogen, phosphorus, and potassium are essential nutrients for the growth and development of higher plants. They are components of various enzymes that participate in many physiological and metabolic processes and significantly impact the development and quality of plants. In this study, after grafting the nitrogen-fixing bacterial strains, it was found that nitrogen absorption by the plant could promote the simultaneous absorption of phosphorus and potassium, thus promoting seedling growth. This may be due to the increase in nitrogen content, which promotes the metabolism of plants, leading to greater absorption of both phosphorus and potassium as well as ATP synthesis, thus promoting plant growth and development [49]. After inoculation of the three bacterial fertilizers, the *A. mongolicus* biomass and contents of the three effective components increased compared with the control. After the bacterial fertilizer application, the plant height, root length, and root dry weight of *A. mongolicus* all increased, and with the fastest growth seen from the July stage to the August stage, the August stage to the September stage. The effects of the nitrogen-fixing bacteria on growth were the most obvious, with the dry weight of the aerial part initially rising and then dropping, reaching a peak during the August period. As the leaves begin to decay after the August period, the dry weight of the above-ground part of the plant decreases more rapidly. The three effective components of *A. mongolicus* all showed increased accumulation, with the greatest accumulation seen from the growth period to the July period. The accumulation of *A. mongolicus* flavonoids and saponins decreased slightly from the August stage to the September stage, which may be due to the consumption of material and energy during fruit formation in this period. Plants accumulate energy and secondary metabolites to prepare for the winter. *A. mongolicus* polysaccharides continued to accumulate before the September period, reaching a maximum during the September period, after which accumulation declined from the September period to the October period. This may be due to the conversion of polysaccharides into other secondary metabolites by plants over the winter.

The mixed inoculation of three kinds of bacteria can significantly change the structure of bacterial flora in rhizosphere soil, which may be the main factor in promoting the growth and accumulation of active components of *A. mongolicus*. The Shannon index showed that the diversity of bacterial community increased significantly with the growth of plants, and the increase was more significant at bacterial agent inoculation treatment. This is consistent with previous reports [50]. However, in this experiment, this difference was insignificant between the treatment and control groups. However, the PCoA analysis results showed significant differences in bacterial community structure between the two groups. This suggests that the strains introduced with the inoculation can significantly affect the composition of the local flora.

The relative abundance of rhizosphere soil bacterial community shows four predominant phyla, Actinobacteriota, Proteobacteria, Acidobacteriota, and Chloroflexi. The bacterial community structure was changed after bacterial fertilizer inoculation. In this study, Proteobacteria increased significantly after inoculation compared with the control group. Proteobacteria is considered to play an important role in biological nitrogen fixation, carbon utilization, and promoting plants to resist environmental stress [51]. Actinobacteriota and Chloroflexi changed little among the groups, indicating that they played an important role in maintaining the stability of bacterial communities in the rhizosphere.

## Conclusions

In this study, we isolated three strains of nitrogen-fixing bacteria (J1, J2, and G4) from root tissue and rhizosphere soil of *A. mongolicus*, which have the advantage of high nitrogen fixation efficiency, good plant growth promotion, and a lack of antagonism to each other. These strains were mixed to form a liquid bacterial fertilizer. Through field experiments, it was found that this bacterial fertilizer significantly promoted plant growth and the main medicinal component accumulation of *A. mongolicus.* Meanwhile, the inoculation of nitrogen-fixing bacterial fertilizer can change the structure of the rhizosphere microbial community and significantly increase the relative abundance of Proteobacteria. These findings provide a basis to apply these nitrogen-fixing bacteria as a good PGRP agent for future scientific cultivation of *A. mongolicus*.

## Data Availability

The data presented in this study are deposited in NCBI (accession numbers: OP297390-OP297397 and PRJNA873723). Further inquiries can be directed to the corresponding author.
